# Topology Optimization of Additively Manufactured Adherends for Increased Adhesive Bond Strength

**DOI:** 10.3390/ma18102170

**Published:** 2025-05-08

**Authors:** Michael Ascher, Ralf Späth

**Affiliations:** Institute for Design and Production Engineering, University of the Bundeswehr Munich, Werner-Heisenberg-Weg 39, 85577 Neubiberg, Germany

**Keywords:** additive manufacturing, powder bed fusion, adhesive bonding, topology optimization, bond strength, finite element method, composite

## Abstract

The limited build space of additive manufacturing (AM) machines constrains the maximum size of AM components, while manufacturing costs rise with geometric complexity. To enhance value and overcome size limitations, it can be more efficient to join non-AM and AM components to meet the requirements by means of a hybrid structure. Adhesive bonding is particularly suitable for such joints, as it imposes no constraints on the joining surface’s geometry or the adherend’s material. To ensure structural integrity, it is conceivable to exploit the design freedom underlying AM processes by optimizing the topology of the AM component to stress the adhesive layer homogeneously. This study explores the feasibility of this concept using the example of an axially loaded single-lap tubular joint between a carbon fiber-reinforced composite tube and an additively manufactured laser-based powder-bed-fusion aluminum alloy sleeve. The sleeve topology was optimized using the finite element method, achieving a 75 %P reduction in adhesive stress increase compared to a non-optimized sleeve. Due to the pronounced ductility of the two-component epoxy-based adhesive, the static bond strength remained unaffected, whereas fatigue life significantly improved. The findings demonstrate the feasibility of leveraging AM design freedom to enhance adhesive joint performance, providing a promising approach for hybrid structures in lightweight applications.

## 1. Introduction

Additive manufacturing (AM) processes, such as laser-based powder-bed fusion of metals (PBF-LB/M), enable the realization of complex topologies with variable stiffness and internal cavities, which can significantly enhance structural performance [1,2]. However, with increasing geometric complexity, manufacturing costs rise due to the need for additional support structures, rework, and longer production times [3]. Additionally, the limited build space of AM machines constrains the maximum size of a single AM component that is manufacturable, affecting the feasibility of AM for various applications. To overcome these limitations and reduce manufacturing costs, part separation and subsequent joining of AM subcomponents can be an effective strategy [4,5]. Moreover, the freedom of design underlying AM processes is often needed only in specific regions of a structure. Thus, it can make sense to enhance the structure with non-AM components (i.e., fiber-reinforced composites) by means of a hybrid structure [6,7]. Adhesive bonding is a particularly promising joining technology in this context, as it adds minimal weight and does not restrict the geometry of the joining surfaces or the material of the adherends [8,9,10,11].

To ensure structural integrity, the adhesive joint design plays a crucial role [12,13]. This entails ensuring a sufficiently large adhesive surface area and preferably subjecting the adhesive to shear rather than peel stress (tensile stress perpendicular to the adhesive surface) [12,13,14]. Adhesive joint designs can roughly be categorized into butt joints and lap joints. In butt joints, the substrates are simply joined end-to-end at their front surfaces. Due to the comparably small adhesive surface area and the occurrence of large peel stresses, this design is less common in structural applications [15]. In lap joints, the joining surfaces of the substrates are overlapping and therefore provide an arbitrarily large adhesive surface area. Disadvantageous is the additional weight due to the material overlap, as well as the eccentric load application in combination with finite adherend stiffness, which causes pronounced adhesive stress increases at the overlap ends [16]. As the stress increases initiate failure [17], it is conceivable to increase the bearable load of the joint (bond strength) while maintaining a constant adhesive surface area by homogenizing the adhesive stress distribution and shifting the adhesive stress state towards pure shear through optimizing the geometry of the adherends.

Finite element (FE)-based genetic algorithm (GA) and topology optimization (TOP) approaches have already been applied to enhance bond strength in rectangular [18,19] and tubular [12] single-lap joints (SLJs). These studies employ either a continuum mechanics approach with energy- or stress-based failure criteria or a fracture mechanics approach utilizing cohesive zone modeling (CZM) and a damage-based failure criterion. While previous TOP procedures neglect adhesive plasticity in the corresponding constitutive response, an essential factor in strength assessment [20,21], the GA approach accounts for debonding through a traction-separation law and considers adhesive plasticity via a Johnson–Cook plasticity model, making it the most suitable method in terms of strength assessment and failure prediction. However, this approach is computationally expensive and, as a result, fails to leverage the extensive design flexibility offered by additive manufacturing. Although all procedures demonstrate significant potential for increased bond strength, previous studies lack experimental validation.

Therefore, in this study, a computationally efficient solid isotropic material with penalization (SIMP) algorithm [22] and implicit FE analysis are used to optimize the topology of an additively manufactured laser-based powder-bed-fusion aluminum alloy AlSi10Mg (PBF-LB/M/AlSi10Mg) sleeve, which is part of an axially loaded single-lap tubular joint (SLTJ) adhesively bonded to an inner carbon fiber-reinforced composite (CFRC) tube using a highly ductile two-component (2C) structural adhesive based on epoxy resin (Scotch-Weld DP490, 3M Deutschland, Neuss, Germany). This adhesive was chosen because it is specifically formulated for bonding of metal and composite materials and is widely used in industrial applications due to its facile processability and high strength characteristics. The joint is modeled using a continuum mechanics approach, where adhesive plasticity is accounted for by means of a multilinear elastoplastic material model. To increase the bond strength of the joint, the element density of the PBF-LB/AlSi10Mg sleeve is iteratively adjusted to achieve homogeneous adhesive shear stress. The optimum sleeve topology found is redesigned considering manufacturing constraints of the PBF-LB/M additive manufacturing process. To quantify the resulting adhesive stress state, non-linear FE analysis is conducted at different tensile loads with tubular joints featuring optimum, redesigned, and non-optimized cylindrical reference sleeves. Subsequently, redesigned and cylindrical reference sleeves are manufactured and adhesively bonded to CFRC tubes to experimentally quantify the difference in bond strength by means of static tensile and fatigue testing.

The main objective of this study is to investigate the extent to which the geometric design freedom offered by additive manufacturing can contribute to improving the bond strength of an axially loaded SLTJ between a CRFC tube and PBF-LB/AlSi10Mg sleeve, when the topology of the sleeve is subjected to FE-based SIMP optimization. Distinct features of this investigation include the incorporation of adhesive plasticity into the optimization process, as well as experimental strength validation.

## 2. Materials and Methods

**Figure 1:**
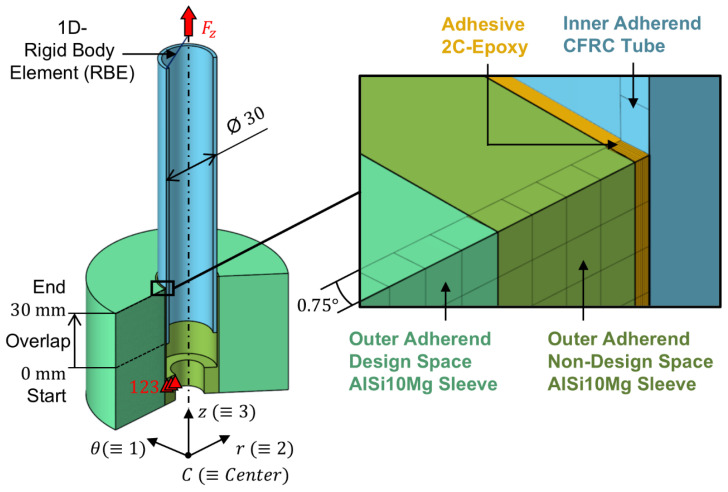
Finite element model (components, analysis coordinate system, and boundary conditions) for SIMP TOP with respect to homogeneous adhesive shear stress.

The axially loaded SLTJ subjected to implicit FE-based TOP comprises an inner adherend, which is a roll-wrapped unidirectional CFRC tube having an outer diameter of 30.0 mm and a wall thickness of 2.0 mm, as well as an outer adherend, which is an additively manufactured PBF-LB/M/AlSi10Mg sleeve having an inner diameter of 30.2 mm. The adherends are joined by an interstitial layer of a two-component epoxy-based (2C-Epoxy) structural adhesive (Scotch-Weld DP490, 3M Deutschland, Neuss, Germany), each having a thickness of 0.1 mm. This approach assumes ideal dimensional accuracy and perfect concentric alignment of the adherends. The outer adherend is divided into the non-design space (NDS) having an outer diameter of 32.2 mm and the design space (DS) having an outer diameter of 100.0 mm. During the optimization process, the element density of the DS is the variable to be altered to meet the objective function of the SIMP optimization (Figure 1).

The overlap between the inner and outer adherend is 30.0 mm, leading to an adhesive surface area of $A_{Adh}=2827.4\;{\mathrm{mm}}^{2}$. To minimize computational effort, only a 0.75° segment of the rotationally symmetric joint was discretized using CHEXA20 second-order solid elements (outer adherend and adhesive), and CQUAD8 second-order shell elements (inner adherend), which were assigned to a cylindrical analysis coordinate system. To enforce rotational symmetry, the azimuthal displacement of elemental nodes at the symmetry faces was constrained to θ=0. The adhesive layer is represented by eight elements in the radial direction (r) and 120 elements in the longitudinal direction (z), resulting in element edge lengths of 0.0125 mm (radially) and 0.25 mm (longitudinally). The average element edge length in the DS of the AlSi10Mg sleeve is 0.35 mm. The contacts between adjacent components are modelled continuously by merging coincident elemental nodes at the component interfaces, thereby assuming perfect adhesion between the adhesive and the adherends and neglecting the influence of surface roughness. To replicate the boundary conditions of the pursuing tensile tests, rigid clamping is applied by fixing all degrees of freedom at nodes on the top surface of the clamping shoulder (bolt head seating according to the tensile test setup detailed below), and a longitudinal tensile force $F_{z}$ is introduced at nodes on the top surface of the CFRC tube using a one-dimensional rigid body element (1D-RBE). As part of the TOP process, the longitudinal tensile force was set to $F_{z,1}=235.6\;N$. Given the reduced adhesive surface area of $A_{Adh,0.75{}^{\circ}}=5.9\;{\mathrm{mm}}^{2}\;$of the 0.75° segment considered, this results in a nominal adhesive shear stress of $\tau_{nom,1}=F_{z,1}/A_{Adh,0.75{}^{\circ}}=40\;\mathrm{MPa}$. The total number of degrees of freedom in the FE model amounts to 564,670.

The material parameters utilized for modeling the structural mechanical behavior of the components are shown in Table 1.

As the von Mises equivalent stress in the AlSi10Mg sleeve must not exceed the yield strength of $R_{e}=227\;\mathrm{MPa}$, it is reasonable to define only linear-elastic material parameters. Neglecting the build-direction-induced anisotropy intrinsic to the PBF-LB/M process is permissible, as the material parameters defined in Table 1 were determined from vertically oriented tensile samples [23], consistent with the intended build direction of the AlSi10Mg sleeves. The CFRC tube exhibits linear-elastic material behavior up to the tensile strength of $R_{||}^{+}=1400\;\mathrm{MPa}$ [24]. Considering a nominal tensile stress in the CFRC tube of $\sigma_{T}=643\;\mathrm{MPa}$ (corresponding to $F_{z,1}=235.6\;N$), the constitutive response of the CFRC tube is fully characterized by specifying linear-elastic material parameters. The CFRC tube laminate is composed of ten stacked unidirectional plies, each having a thickness of 0.2 mm. The fibers extend parallel to the longitudinal (z) direction of the tube. By modeling the laminate on a ply basis, the layer stack and fiber orientation can be represented by single shell elements. This requires specifying the orthotropic material parameters of a single unidirectional ply, along with the orientation and order of the plies. The material parameters provided in Table 1 refer to a unidirectional ply with high tenacity (HT) fibers and a fiber content of 60% according to [24]. Since the 2C-Epoxy adhesive exhibits non-linear stress–strain behavior far below the ultimate strength of $\sigma_{2C,Ult}=31.26\;\mathrm{MPa}$, pure consideration of elasticity is insufficient for structural–mechanical analysis [20,21]. To account for the adhesive’s plasticity, a multilinear elastoplastic material model is employed in [25]. In doing so, the technical stress–strain curve obtained from static tensile tests using adhesive bulk samples according to [26] is approximated using multiple regression lines, and the deflection points of the locally linearized stress–strain curve serve as input parameters for the material model. By assuming elastic and plastic isotropy, the structural–mechanical behavior of the 2C-Epoxy adhesive is fully characterized by additionally specifying the shear modulus G, which was determined through torsion testing of butt-bonded hollow cylinders [25].

The optimization function considered by the SIMP algorithm is defined by an optimization target and optimization constraints [22]. As FE-based analysis of an axially loaded SLTJ using CZM shows that bonding failure, following a bi-linear traction-separation law, correlates with the maximum first principal stress adjacent to the bonding interface [12], the optimization target is set to minimize the first principal stress $\sigma_{1,max}$ within the adhesive component. Considering a reference value for the first principal stress of $\sigma_{1,ref}=\tau_{nom,1}=40\;\mathrm{MPa}$, pure adhesive shear and minimum peel stress are aimed for. Optimization constraints regarding the aluminum sleeve (DS and NDS) include a maximum von Mises equivalent stress of $\sigma_{vMises,\;max}=R_{e}=227\;\mathrm{MPa}$ and a mass reduction of 50% in the DS. To meet the objective function criteria, the SIMP algorithm iteratively adjusts the element density (ED) of each individual element in the DS of the PBF-LB/AlSi10Mg sleeve. The ED can vary in the range of 0≤ED≤1, where ED=0 corresponds to no material and ED=1 corresponds to solid material. Since the penalization parameter of the SIMP algorithm is set to p=2, intermediate element densities (0<ED<1) are penalized, promoting a clear distinction between solid and void regions. The optimization process converges when the relative change in the objective function between two consecutive iterations falls below the threshold of 0.5%.

To quantify the resulting adhesive stress state, the optimum sleeve topology is FE-analyzed at tensile loads of $F_{z,1}=235.6\;N$ and $F_{z,2}=58.9\;N$, corresponding to nominal adhesive shear stresses of $\tau_{nom,1}=F_{z,1}/A_{Adh,0.75{}^{\circ}}=40\;\mathrm{MPa}$ (nonlinear) and $\tau_{nom,2}=F_{z,2}/A_{Adh,0.75{}^{\circ}}=10\;\mathrm{MPa}$ (linear), and the first principal stress is evaluated for all 120 elements representing the adhesive layer along the overlap (z-direction) at adhesive mid-thickness (in the fifth of eight element rows representing the adhesive layer in the positive r-direction). For classification, this result is contrasted with the course of the first principal stress induced by a redesigned sleeve and a non-optimized cylindrical reference sleeve having an outer diameter of 50 mm. The redesigned sleeve is based on the optimum topology while considering manufacturing constraints related to the PBF-LB/M process. The redesign was generated based on the contour plot of the optimum ED using computer-aided design (CAD) software (CATIA V5-6R2016, Dassault Systèmes, Vélizy-Villacoublay, France). In doing so, the contours depicted were resketched (ED<0.7  neglected) and abstracted to comply with a minimum overhang angle of 35° and a minimum wall thickness of 1.0 mm [2,27]. Subsequently, the sketch was rotated over an angle of 0.75° to form a solid body, which served as a basis for the ensuing FE analysis. Both the FE-based stress analyses and the TOP procedure were conducted using commercially available FE software (HyperWorks 2021.2, Altair Engineering, Troy, MI, USA) with the OptiStruct solver.

To experimentally quantify the bond strength of the redesigned and reference sleeves, static tensile and fatigue tests were performed using a total of 24 bonded tensile samples. These include four redesigned and four cylindrical reference sleeves for conducting static tensile tests, as well as eight redesigned sleeves and eight cylindrical reference sleeves for conducting fatigue tests. The sleeves were manufactured using a DMG MORI LASERTEC 30 SLM 2nd Generation (Bielefeld, Germany) PBF-LB/M additive manufacturing machine. The associated manufacturing parameters are depicted in Table 2.

**Figure 2:**
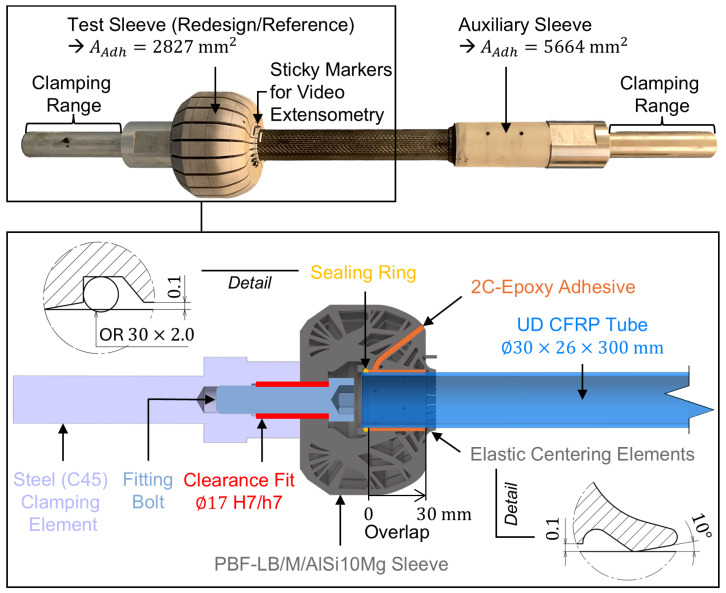
Schematic of a test sleeve forming part of a bonded sample for conducting tensile tests (**bottom**), and photograph of an entire bonded sample with an auxiliary sleeve to clamp the CFRC tube in the test machine (**top**).

Commercially available AlSi10Mg powder (EN-AC 43000, as specified in [28]) with a particle size between 20 µm and 60 µm was used throughout. To suppress influences on the surface roughness caused by varying component orientation and to ensure consistency with the material parameters provided in Table 1, the AlSi10Mg sleeves were manufactured vertically oriented (longitudinal axis perpendicular to the build plate). The redesigned sleeve features 20 equidistant radially symmetric slots with a slot opening angle of 3° to facilitate the removal of residual powder from the internal cavities. The maximum and minimum inner diameters of the 24 sleeves were determined to be $d_{max}=30.299\;\mathrm{mm}$ and $d_{min}=30.227\;\mathrm{mm}$ by optical measurement using a 3D laser scanning microscope (Keyence, VK-X 3000). Commercially available roll-wrapped unidirectional CFRC tubes with nominal dimensions of ∅30×26×300 mm were used as inner adherends. They offer an HT fiber content of 60% and a ground outer surface finish. The tubes’ maximum and minimum inner diameters were determined to be $d_{max}=30.086\;\mathrm{mm}$ and $d_{min}=30.053\;\mathrm{mm}$ using a digital outside micrometer (Absolute Digimatic 2, Mitutoyo, Kawasaki, Japan). A 2C-Epoxy structural adhesive (Scotch-Weld DP490, 3M Deutschland, Neuss, Germany) was used to adhesively bond the inner and outer adherend. By assuming perfect concentric alignment of the adherends, based on the measured inner sleeve and outer tube diameters, the actual adhesive layer thickness ranges from $t_{min}=0.114\;\mathrm{mm}$ to $t_{max}=0.167\;\mathrm{mm}$ with an arithmetic mean of $\bar{t}=\left(0.124\pm 0.021\right)\;\mathrm{mm}$. Figure 2 shows a schematic of a test sleeve (bottom) and an exemplary entire tensile test sample (top).

The preceding steps up to the completion of the bonded tensile test sample were as follows: After separating the de-powdered sleeves from the build plate using a band saw, the bottom surface was faced on a lathe. Then, the adhesive surface of the sleeves was grid-blasted with corundum (F200), cleaned in an ultrasonic bath with isopropanol, rinsed, and dried. Subsequently, the sleeves were bolted to steel clamping elements using a special fitting bolt to ensure concentric alignment. Next, a sealing ring (OR 30×2.0) was inserted into a designated groove within the sleeve, followed by the CFRC tube, which is concentrically aligned with the sleeve by the sealing ring at the bottom and elastic centering elements integrated at the top of the sleeve.

The adhesive surface of the CFRC tube was mechanically treated using abrasive fleece (Scotch-Brite CF-HP 7447, 3M Deutschland, Neuss, Germany), followed by cleaning with isopropanol before being inserted into the sleeve. No bonding agent was used. Then, the adhesive was injected, filling the adhesive fill gap between the adherends according to [29,30]. For hardening, the samples were stored in a climatic chamber for two hours at 65 °C and then conditioned to the standard climate (23 °C/50%) for at least 24 h. For clamping the CFRC tube in the test machine, a cylindrical auxiliary sleeve overlapping the tube by 60 mm was employed. Given the doubled adhesive surface area relative to the test sleeve, failure of the auxiliary sleeve’s adhesive bond is highly improbable.

The static tensile tests were carried out using a 600 kN servo-hydraulic tensile-testing machine (Schenck-Trebel, Carl Schenck, Darmstadt, Germany). The bonded samples were fixed over a clamping range of 100 mm using wedge grips. To steadily increase the load on the joint, the testing machine was operated in a displacement controlled manner with a constant test speed of 1.5 mm/min [31]. The test results are documented in the form of nominal shear stress versus machine stroke diagrams, from which the maximum measured nominal adhesive shear stress (static bond strength) is determined by relating the maximum force measured to the sleeve’s adhesive surface area, $A_{Adh}=2827.4\;{\mathrm{mm}}^{2}$. A video extensometer (RTSS, LIMESS, Krefeld, Germany) was used to measure the z-strain between two high-contrast sticky markers applied to the lateral surfaces of the CFRC tube and the AlSi10Mg sleeves (see Figure 2, top). To validate the FE model, the corresponding z-strain was determined by means of nonlinear FE analysis by evaluating the difference in z-displacement between elemental nodes positioned at the same locations as the contrast lines of the sticky markers. Up to a nominal shear stress of 30 MPa, the stress–strain response obtained from tensile testing aligns well with the results of the nonlinear FE analysis for joints featuring cylindrical reference sleeves, supporting the validity of the FE model (see Appendix A).

The fatigue strength of the joints was determined through pulsating tensile fatigue tests using a 150 kN SincoTec Power Swing (Clausthal-Zellerfeld, Germany) electromechanical resonance testing machine, where eight bonded tensile samples with redesigned test sleeves were subjected to cyclic tensile loading with sinusoidal progression at nominal shear stress amplitudes of $\tau_{nom,A1}=7.5\;\mathrm{MPa}$ or $\tau_{nom,A2}=5.0\;\mathrm{MPa}$, while eight bonded tensile samples with reference test sleeves were subjected to nominal shear stress amplitudes of $\tau_{nom,A3}=6.4\;\mathrm{MPa}$ and $\tau_{nom,A4}=4.3\;\mathrm{MPa}$. All tests were conducted with identical stress ratios of R=0 and test (resonance) frequencies ranging from 49 Hz to 54 Hz. Four tensile samples were subjected to each stress amplitude, and the number of load cycles endured was counted until the maximum endurable stress of the test sleeve’s adhesive bond dropped below 95% of the respective maximum stress $\tau_{max}=2\tau_{nom,A}$. The load amplitudes were determined during preliminary tests to ensure that the number of endurable load cycles ranges from $N_{min}=10^{4}$ to $N_{max}=10^{7}$. Accordingly, the results can be presented in a Wöhler (S-N) diagram, where a finite-life fatigue curve for each sleeve is evaluated at a 50% failure probability using the horizontal method [32].

## 3. Results

### 3.1. Numerical Results

**Figure 3:**
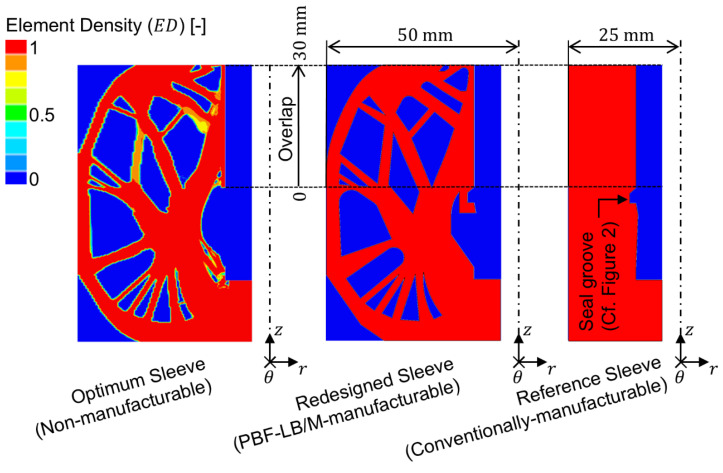
Contour plot of element densities applying to the optimum sleeve, redesigned sleeve, and cylindrical reference sleeve (1≡ solid and 0≡ void).

Figure 3 depicts the topology (DS and NDS) of the optimum sleeve (as a direct result of the FE-based TOP process), the redesigned sleeve (based on the optimum sleeve, abstracted in favor of PBF-LB/M manufacturability), and the cylindrical reference sleeve in a sectional view by means of a contour plot.

**Figure 4:**
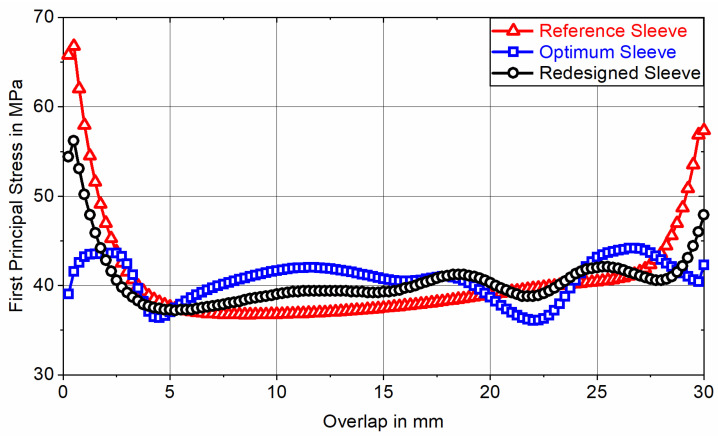
First principal stress at adhesive mid-thickness as a function of the overlap for nonlinear FE analysis of axially loaded tubular joints between a CFRC tube and sleeves with different topologies (according to Figure 3), exposed to a nominal adhesive shear stress of $\tau_{nom,1}=40\;\mathrm{MPa}$.

With a total mass of $m_{0.75{}^{\circ}}=1.58\;g$, the 0.75° segment of the optimum sleeve achieves a mass reduction of approximately 47% compared to the initial weight of the DS, while exhibiting a maximum von Mises stress of $\sigma_{vMises,\;max}=224\;\mathrm{MPa}$ ($<R_{e})$. Due to the abstraction of the optimum sleeve’s contour in favor of PBF-LB/M manufacturability, the redesigned sleeve exhibits an average deviation from the optimum element density of 5.8%. This results in a minor increase in total mass of $m_{0.75{}^{\circ}}=1.63\;g$ and a slight decrease in maximum von Mises stress to $\sigma_{vMises,\;max}=217\;$MPa. The impact of this optimization degradation on the adhesive stress state is evaluated by comparing the first principal stress distribution at adhesive mid-thickness for nonlinear FE analysis of joints featuring the optimum sleeve and the redesigned sleeve for loading with a nominal adhesive shear stress of $\tau_{nom,1}=40\;\mathrm{MPa}$. To put these results into perspective, they are compared to the first principal stress distribution determined for nonlinear FE analysis of joints featuring a non-optimized reference sleeve (Figure 4).

**Figure 5:**
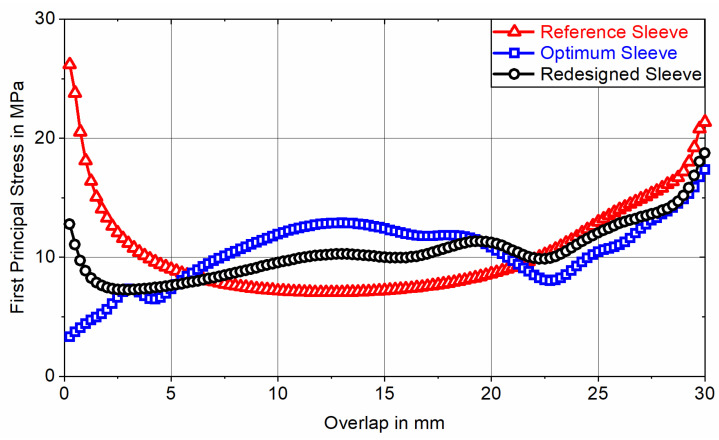
First principal stress at adhesive mid-thickness as a function of the overlap for linear FE analysis of axially loaded tubular joints between a CFRC tube and sleeves with different topologies (according to Figure 3), exposed to a nominal adhesive shear stress of $\tau_{nom,2}=10\;\mathrm{MPa}$.

With a maximum first principal stress of $\sigma_{1,max}=44.2\;\mathrm{MPa}$, the adhesive bond of the optimum sleeve results in a maximum adhesive stress increase of 10% in relation to the reference value of $\sigma_{1,ref}=\tau_{nom,1}=40\;\mathrm{MPa}$. Compared to the cylindrical reference sleeve, which has a maximum adhesive stress increase of 67% ($\sigma_{1,max}=66.8\;\mathrm{MPa}\;\mathrm{and}\;\epsilon_{1,max}=37\%$), this is a significant improvement. Looking at the course of the first principal stress for the redesigned sleeve, a maximum adhesive stress increase of 40% ($\sigma_{1,max}=56.2\;\mathrm{MPa}$) applies. Although the maximum stress increase is reduced by 27%P compared to the reference sleeve, the optimization degradation in favor of PBF-LB/M manufacturability results in a 30%P higher maximum stress increase compared to the optimum sleeve. Consequently, the optimum sleeve effectively transfers the load through adhesive shear stress, whereas the proportion of load transferred via adhesive peel stress progressively increases from the redesigned sleeve to the reference sleeve. Due to the adhesive’s non-linear stress–strain-behavior, it is to be expected that FE analysis at different nominal loads will result in varying adhesive stress increases. Figure 5 depicts the course of the first principal stress at adhesive mid-thickness along the overlap when the joints are subjected to a nominal adhesive shear stress of $\tau_{nom,2}=10\;\mathrm{MPa}$.

By exhibiting maximum first principal stresses of $\sigma_{1,max}=17.4\;\mathrm{MPa}$ (Optimum Sleeve), $\sigma_{1,max}=18.7\;\mathrm{MPa}$ (Redesigned Sleeve), and $\sigma_{1,max}=26.2\;\mathrm{MPa}$ (Reference Sleeve), the corresponding maximum adhesive stress increases rise from 10% at $\tau_{nom,1}=40\;\mathrm{MPa}$ to 74% at $\tau_{nom,2}=10\;\mathrm{MPa}$ (Optimum Sleeve), 40% at $\tau_{nom,1}=40\;\mathrm{MPa}$ to 87% at $\tau_{nom,2}=10\;\mathrm{MPa}$ (Redesigned Sleeve), and 67% at $\tau_{nom,1}=40\;\mathrm{MPa}$ to 162% at $\tau_{nom,2}=10\;\mathrm{MPa}$ (Reference Sleeve). The maximum first principal strain reaches $\epsilon_{1,max}=1.8\%$ for the joint featuring the reference sleeve. Since $\epsilon_{1,max}<5\%$ applies, linear FE analysis is permissible [33]. Due to less adhesive plasticization at lower loads, local stress increases can only be reduced to a limited extent by yielding. This effect is most pronounced in the adhesive bond of the reference sleeve, where the maximum stress increase rises by 95%P when comparing higher to lower loads. The adhesive bond of the optimum sleeve shows a slightly lower increase of 64%P between higher and lower loading, while the redesigned sleeve exhibits the smallest increase in maximum stress at 47%P. This smaller increase induced by the redesigned sleeve can be attributed to its topology already deviating from the optimum due to abstraction in favor of PBF-LB/M manufacturability. In contrast, the optimum sleeve topology is optimized towards loading with $\tau_{nom,1}=40\;\mathrm{MPa}$ and thus no longer aligns effectively with the stiffness requirements at divergent loading (i.e., $\tau_{nom,2}=10\;\mathrm{MPa})$. As a result, the maximum adhesive stress increase induced by the redesigned sleeve exceeds that of the optimum sleeve for reduced loading at $\tau_{nom,2}=10\;\mathrm{MPa}$ by only 10%P (30%P at $\tau_{nom,1}=40\;\mathrm{MPa}$), while falling 75%P below the maximum stress increase induced by the reference sleeve (27%P at $\tau_{nom,1}=40\;\mathrm{MPa}$). In relation to actual bond strength, this indicates that the service life of the redesigned sleeve should exceed that of the reference sleeve, with the difference becoming more pronounced at lower nominal shear stress amplitudes. In contrast, the gain in static tensile strength is expected to remain limited, as local adhesive yielding continues to alleviate stress increases.

### 3.2. Experimental Results

#### 3.2.1. Static Strength

**Figure 6:**
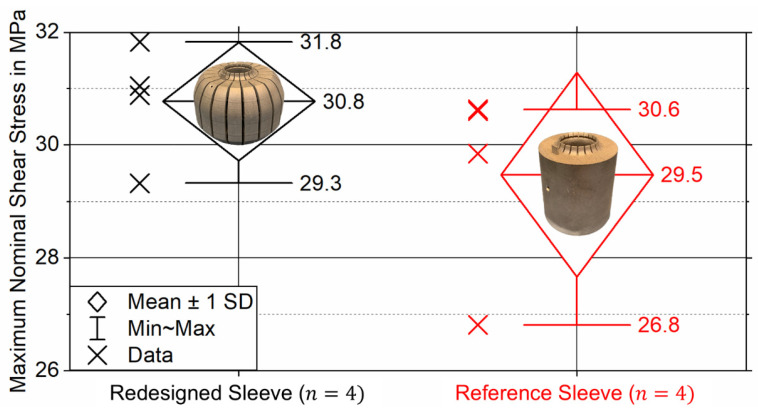
Maximum nominal shear stress for static tensile testing of eight SLTJs between CFRC tubes and AlSi10Mg sleeves with different topologies (according to Figure 3).

Figure 6 depicts the maximum nominal adhesive shear stress for static tensile testing of eight tensile test samples featuring four PBF-LB/M-manufacturable redesigned sleeves and four conventionally manufacturable (cylindrical) reference sleeves, single-lap bonded to unidirectional CFRC tubes.

Assuming a Gaussian normal distribution, the mean values (n=4) of the maximum nominal adhesive shear stress and standard deviations (SDs) of the adhesive bonds featuring redesigned and reference sleeves can be evaluated as ${\bar{\tau }}_{max,Red}=\left(30.8\pm 0.9\right)\;\mathrm{MPa}$ and ${\bar{\tau }}_{max,Ref}=\left(29.5\pm 1.6\right)\;\mathrm{MPa}$. To determine if the mean values vary significantly (significance level of 5%, i.e., α=0.05), an independent one-tailed homoscedastic Student’s *t*-test was conducted. The p-value comparing the mean values is calculated as p=0.13. Since p>α, it can be concluded that the difference in static bond strength between the redesigned and reference sleeves is insignificant.

#### 3.2.2. Fatigue Strength

**Figure 7:**
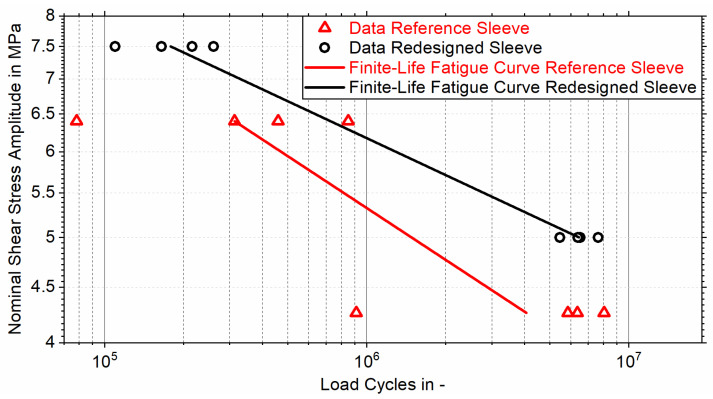
Results of pulsating (*R* = 0) tensile testing of tubular joints between CFRC tubes and AlSi10Mg sleeves with different topologies (according to Figure 3), and finite-life fatigue curves for a failure probability of 50%.

Figure 7 depicts the results of pulsating (R=0) tensile fatigue tests conducted at nominal shear stress amplitudes of $\tau_{nom,A1}=7.5\;\mathrm{MPa}$ and $\tau_{nom,A2}=5.0\;\mathrm{MPa}$ (redesigned sleeve), and $\tau_{nom,A3}=6.4\;\mathrm{MPa}$ and $\tau_{nom,A4}=4.3\;\mathrm{MPa}$ (reference sleeve), using four bonded tensile test samples per load amplitude.

The evaluation of the test results for the tubular joints featuring non-optimized reference sleeves, based on a logarithmic normal distribution with a failure probability of 50%, yields a slope exponent of the corresponding finite-life fatigue curve of $k_{50\%,Ref}=6.3$. The corresponding service life is $N_{50\%,3}=312,673$ at a load amplitude of $\tau_{nom,A3}=6.4\;\mathrm{MPa}$ and $N_{50\%,4}=4,065,332$ at a load amplitude of $\tau_{nom,A4}=4.3\;\mathrm{MPa}$. An identical evaluation of the tubular joints featuring redesigned sleeves results in a slope exponent of the corresponding finite-life fatigue curve of $k_{50\%,Red}=8.8$, with a service life of $N_{50\%,1}=178,747$ at a load amplitude of $\tau_{nom,A1}=7.5\;\mathrm{MPa}$ and $N_{50\%,2}=6,455,323$ at a load amplitude of $\tau_{nom,A2}=5.0\;\mathrm{MPa}$. By extrapolating the finite-life fatigue curves towards higher load amplitudes, an intersection point is identified at $\tau_{nom,0}=8.9\;\mathrm{MPa}$ and $N_{50\%,0}=39,193$. At this load amplitude, the tubular joints featuring redesigned and reference sleeves exhibit similar service lives. As the load amplitude decreases, the service life of the redesigned sleeve increases more rapidly than that of the reference sleeve due to the higher slope exponent. Consequently, at a nominal shear stress amplitude of $\tau_{nom,A2}=5.0\;\mathrm{MPa}$, the redesigned sleeve achieves a service life that exceeds that of the reference sleeve by 336%. By utilizing the Basquin equation, the service lives of reference sleeves corresponding to failure probabilities of 10% ($N_{10\%,2}$), 50% ($N_{50\%,2}$), and 90% ($N_{90\%,2}$) at a load amplitude of $\tau_{nom,A2}=5.0\;\mathrm{MPa}$ can be determined. Based on these values, the standard deviations of the service lives of joints featuring both reference and redesigned sleeves can be calculated at the same load amplitude. By comparing the t-value (t=4.5) obtained from Welch’s *t*-test with the critical t-value $t_{crit}=2.3$ for a 5% significance level, determined using the Welch–Satterthwaite equation, it can be concluded that there is a significant difference in service life between redesigned and reference sleeves.

## 4. Discussion

The results of the FE stress analysis of the optimum and redesigned sleeves indicate that, due to the abstraction of the optimum sleeve’s contours in favor of PBF-LB/M manufacturability, the resulting redesigned sleeve demonstrates reduced performance in achieving a homogeneous adhesive shear stress distribution compared to the optimum sleeve. As the advantages of the optimum sleeve topology are associated with the nominal adhesive shear stress considered during the optimization process, the difference in performance between the optimum and redesigned sleeve topologies decreases for load conditions deviating from the one considered in the optimization process. Upon comparing the results obtained from FE stress analysis of the non-optimized reference sleeve with the redesigned sleeve, it was found that the maximum adhesive stress increase induced by the reference sleeve exceeds that of the redesigned sleeve by 27%P for loading with $\tau_{nom,1}=40\;\mathrm{MPa}$ and by 75%P for loading with $\tau_{nom,2}=10\;\mathrm{MPa}$. This shows that the benefits of the redesigned sleeve in terms of achieving a homogeneous adhesive stress distribution become more pronounced at lower loads, as, for reduced adhesive ductility, the ability to relieve stress increases through adhesive yielding is limited. The significant influence of adhesive plasticity on performance of optimized adherend geometry is also evident when comparing the 34% peak peel stress reduction between the optimum and reference sleeve topologies evaluated in this study to the 63% peak peel stress reduction obtained for TOP of a rectangular SLJ using an energy-based failure criterion in [19]. As in [19], adhesive plasticity was neglected in the corresponding FE analysis; the total peak peel stress reduction between the optimum and reference geometry exceeds that obtained in this study by almost a factor of two.

In the course of static tensile tests using bonded tensile samples featuring redesigned and reference sleeves, no difference in bond strength was demonstrable. The discrepancy between the numerical and experimental results can be attributed to the underestimation of the adhesive’s ductility in the FE model. Once the adhesive’s ultimate strength of $\sigma_{2C,Ult}=31.26\;\mathrm{MPa}$ is exceeded, the final slope of the multilinear stress–strain curve (as presented in Table 1) is extrapolated and, consequently, accounts for all stress–strain conditions beyond the ultimate strength. Assuming perfect plasticity of the adhesive after surpassing its ultimate strength would significantly reduce numerical stress increases for all sleeve topologies. A more accurate representation of the joint’s structural failure behavior could also be achieved by incorporating fracture mechanics into the FE model using CZM. As shown in [12], by implementing cohesive zone elements at the interfaces between adhesive and adherends, their degradation based on a traction–separation law could be captured, resulting in more accurate stress–strain prediction for ductile adhesives at higher loads [34].

Since the adhesive exhibits minimal plasticization during pulsating (R=0) fatigue tests under loading with nominal shear stress amplitudes between $\tau_{nom,A1}=7.5\;\mathrm{MPa}$ and $\tau_{nom,A4}=4.3\;\mathrm{MPa}$, a correlation between the numerical adhesive stress increase and the corresponding service life was captured. The finite-life fatigue curves of tubular joints with redesigned and reference sleeves show that, as the load decreases, adhesive plasticization diminishes, resulting in a fourfold increase in the service life of the redesigned sleeve compared to the reference sleeve at a shared load amplitude of $\tau_{nom,A2}=5.0\;\mathrm{MPa}$.

## 5. Conclusions

The findings emphasize that the effectiveness of adherend TOP with respect to increased bond strength strongly depends on the structural–mechanical behavior of the adhesive and the applied load regime. Incorporating adhesive plasticity in FE-based analysis and optimization is crucial when using ductile adhesives, as the adhesive stress distribution is significantly affected by the material’s ability to locally yield and redistribute stresses. Consequently, the effectiveness of an optimized adherend geometry in reducing adhesive stress concentration depends on the applied load and diminishes for loading conditions deviating from those considered in the optimization process.

Furthermore, the performance of the optimized adherend geometry is reduced by abstracting the topology in favor of manufacturability. In this regard, additive manufacturing presents a key advantage, as it enables designers to minimize optimization degradation through the realization of complex geometries with variable stiffness and internal cavities. Future studies could focus on minimizing optimization degradation by incorporating lattice structures, particularly at critical regions such as the overlap start and end, where adhesive stress increases are most pronounced.

The comparison between experimental and numerical results highlights the necessity of accurately modeling adhesive plasticity and failure behavior within the FE framework, particularly when aiming to improve static bond strength. Future studies should focus on refining the representation of adhesive failure mechanisms by integrating cohesive zone models into the topology optimization (TOP) process. This would enable more reliable predictions of adhesive stresses while simultaneously identifying advanced, high-performance designs.

## Figures and Tables

**Table 1:** Material parameters of the adherends (AlSi10Mg sleeve according to [23] and CFRC tube according to [24]) and the 2C-Epoxy adhesive according to [25].

Parameter	Elastic Modulus *E* in GPa		Shear Modulus *G* in GPa		Poisson’s Ratio *ν* in -	Density *ρ* in g/cm^3^
AlSi10Mg Sleeve (linear isotropic)	66.1		-		0.39	2.66
CFRC Tube (linear orthotropic)	139.4 (||) 8.8 (⊥)		4.6 (||⊥) 3.2 (⊥⊥)		0.29 (||⊥) 0.37 (⊥⊥)	-
2C-Epoxy Adhesive (multilinear isotropic)	1.74		-		0.32	-
Support Points of the Adhesive’s Multilinear Stress–Strain Curve						
Tech. Strain in %	0	0.81	1.50	1.99	2.56	3.59
Tech. Stress in MPa	0	14.1	22.6	26.9	29.8	31.1

**Table 2:** Manufacturing parameters used to fabricate AlSi10Mg sleeves using a DMG MORI LASERTEC 30 SLM PBF-LB/M additive manufacturing machine.

Parameter	Hatch/Contour
Scanning Speed [mm/s]	1500/400
Laser Power [W]	450/280
Focus Diameter [µm]	120/65
Layer Thickness [µm]	50
Hatch/Contour Offset [µm]	170/150
Rotation Angle of Scan Pattern [°]	53
Inert Gas Flow Rate [L/min]	1000
Oxygen Target Value [%]	0.15
Base Plate Heating Temperature [°C]	200
Focus Diameter [µm]	120/65

**Figure A1:**
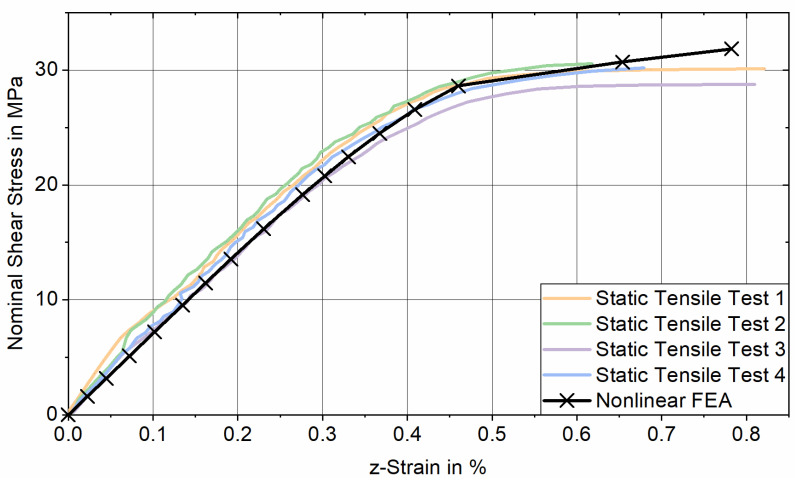
Stress–strain response obtained from static tensile testing of four tensile samples involving reference sleeves and nonlinear FEA of the corresponding joint.

## Data Availability

The original contributions presented in this study are included in the article. Further inquiries can be directed to the corresponding author.

## Abbreviations

The following abbreviations are used in this manuscript:

AM	Additive Manufacturing
PBF-LB/M	Laser-based powder-bed fusion of metals
FE	Finite Element
GA	Generic Algorithm
TOP	Topology Optimization
SLJ	Single-lap joint
CZM	Cohesive Zone Model
SIMP	Solid isotropic material with penalization
SLTJ	Single-lap tubular joint
2C	Two-Component
CFRC	Carbon fiber-reinforced composite
DS	Design Space
NDS	Non-Design Space
RBE	Rigid Body Element
SD	Standard Deviation
CAD	Computer-Aided Design
ED	Element Density
HT	High Tenacity

## References

[1] Pei E., Bernard A., Gu D., Klahn C., Monzón M., Petersen M., Sun T. (2023). Springer Handbook of Additive Manufacturing.

[2] Yang L., Hsu K., Baughman B., Godfrey D., Medina F., Menon M., Wiener S. (2017). Additive Manufacturing of Metals: The Technology, Materials, Design and Production.

[3] Reichwein J., Kirchner E. (2021). Part orientation and separation to reduce process costs in additive manufacturing. Proc. Des. Soc..

[4] Reichwein J., Geis J., Rudolph K., Kirchner E. (2022). Design guidelines for the separation of components to combine the potentials of additive and conventional manufacturing processes. Procedia CIRP.

[5] Ascher M., Späth R. (2024). Additively manufactured 3D micro scarf adhesive joints. Proc. Des. Soc..

[6] How We Build Your Bike. https://www.athertonbikes.com/technology/additive-manufacturing.html.

[7] Ascher M., Späth R., Lachmayer R., Bode B., Kaierle S. (2022). Joining Technology of Additively Manufactured Components: Design Measures for Optimizing the Strength of Adhesively Bonded Joints. Innovative Product Development by Additive Manufacturing 2021.

[8] Cavalcanti D.K.K., Banea M.D., Queiroz H.F.M. (2019). Mechanical Characterization of Bonded Joints Made of Additive Manufactured Adherends. Ann. Dunărea Jos Univ. Galati Fascicle XII Weld. Equip. Technol..

[9] Cavalcanti D.K.K., Medina M., Queiroz H.F.M., Neto J.S.S., Chaves F.J.P., Banea M.D. (2023). Recent Advances in Adhesive Bonding of 3D-Printed Parts and Methods to Increase their Mechanical Performance. Ann. Dunărea Jos Univ. Galati Fascicle XII Weld. Equip. Technol..

[10] Frascio M., Marques E.A.d.S., Carbas R.J.C., da Silva L.F.M., Monti M., Avalle M. (2020). Review of Tailoring Methods for Joints with Additively Manufactured Adherends and Adhesives. Mater.

[11] Campilho R.D.S.G. (2025). Advances in Adhesive Bonding Techniques for 3D-Printed Components. Clareus Sci. Sci. Eng..

[12] Arhore E.G., Yasaee M., Dayyani I. (2021). Comparison of GA and topology optimization of adherend for adhesively bonded metal composite joints. Int. J. Solids. Struct..

[13] Silva L.F.M.d., Öchsner A., Adams R.D. (2018). Handbook of Adhesion Technology.

[14] Banea M.D., Da Silva L.F.M. (2009). Adhesively bonded joints in composite materials: An overview. Proc. Inst. Mech. Eng..

[15] Krittanai C., Honghirun T., Preechasuth B., Nusom Y., Uthaisangsuk V. (2025). Mechanical and failure behaviors of adhesively bonded dissimilar materials joints incorporating bio-inspired morphological irregularities. Int. J. Adhes. Adhes..

[16] Gonçalves D.J.S., Campilho R.D.S.G., Da Silva L.F.M., Fernandes J.L.M. (2014). The Use of the Boundary Element Method in the Analysis of Single Lap Joints. J. Adhes..

[17] Canyurt O.E., Zhang J. (2006). Pre-stressed adhesive strap joints for thick composite sandwich structures. Int. J. Mech. Sci..

[18] Kaufmann M., Vallée T. (2022). Topology optimization of adhesively bonded double lap joints. Int. J. Adhes. Adhes..

[19] Ejaz H., Mubashar A., Ashcroft I.A., Uddin E., Khan M. (2018). Topology optimisation of adhesive joints using non-parametric methods. Int. J. Adhes. Adhes..

[20] da Silva L.F.M. (2008). Modeling of Adhesively Bonded Joints.

[21] da Silva L.F.M., Campilho R.D.S.G. (2012). Advances in Numerical Modelling of Adhesive Joints. SpringerBriefs in Applied Sciences and Technology.

[22] Papadopoulos I.P.A. (2025). Numerical analysis of the SIMP model for the topology optimization problem of minimizing compliance in linear elasticity. Numer. Math..

[23] Sert E., Schuch E., Öchsner A., Hitzler L., Werner E., Merkel M. (2019). Tensile strength performance with determination of the Poisson’s ratio of additively manufactured AlSi10Mg samples. Mater. Werkst..

[24] Schürmann H. (2007). Konstruieren Mit Faser-Kunststoff-Verbunden.

[25] Ascher M., Späth R., Johlitz M., Altenbach H., Hitzler L., Johlitz M., Merkel M., Öchsner A. (2024). Elastoplastic Characterization of a Two-Component Epoxy-Based Structural Adhesive. Lecture Notes on Advanced Structured Materials 2.

[26] (2012). Plastics-Determination of Tensile Properties-Part 2: Test Conditions for Moulding and Extrusion Plastics.

[27] Diegel O., Nordin A., Motte D. (2019). A Practical Guide to Design for Additive Manufacturing.

[28] (2020). Aluminium and aluminium alloys-Castings-Chemical Composition and Mechanical Properties.

[29] Ascher M., Brenner S., Pang G.A., Späth R. (2023). Joining Technology of Additively Manufactured Components: Effects on the Bonding Strength for the Adhesive Application through Inner Channels. Prog. Addit. Manuf..

[30] Ascher M., Pang G.A., Späth R. (2023). Method for the design of additively manufactured inner channels intended for adhesive application. Procedia CIRP.

[31] (2009). Adhesives-Determination of Tensile Lap-Shear Strength of Bonded Assemblies.

[32] (2022). Load Controlled Fatigue Testing-Execution and Evaluation of Cyclic Tests at Constant Load Amplitudes on Metallic Specimens and Components.

[33] Introduction to Nonlinear Finite Element Analysis Using Altair OptiStruct. https://altairuniversity.com/free-ebook-introduction-to-nonlinear-finite-element-analysis-using-optistruct/.

[34] Ramalho L., Campilho R., Belinha J., Da Silva L. (2020). Static strength prediction of adhesive joints: A review. Int. J. Adhes. Adhes..

